# Decision-Making Styles and Managerial Creativity of Nursing Managers in Saudi Arabia: A Multi-Center Cross-Sectional Study

**DOI:** 10.3390/healthcare11121719

**Published:** 2023-06-12

**Authors:** Naheel A. AlAmer

**Affiliations:** Department of Family and Community Medicine, College of Medicine, Imam Abdulrahman Bin Faisal University, Dammam 34224, Saudi Arabia; naalamer@iau.edu.sa

**Keywords:** decision-making styles, rational decision, avoidant decision, dependent decision, creativity, nursing managers, top manager, middle manager, low-level manager

## Abstract

(1) Background: Nursing managers as responsible personnel are required to think outside the box in order to make useful decisions using an appropriate style in a creative manner. This study aims to investigate the relationship between nursing managers’ decision-making styles and managerial creativity. (2) Methods: A multi-center cross-sectional design was used to collect data from 245 managers in five large government hospitals using self-administered questionnaires on managerial creativity and general decision-making styles. (3) Results: A significant relationship was found between rational, avoidant, and dependent styles and total managerial creativity. A positive correlation was present between the rational style and total managerial creativity and a negative correlation was found between avoidant, dependent, and spontaneous styles and total managerial creativity. According to a regression analysis, the rational style has a positive effect on managerial creativity, whereas the dependent and avoidant styles have a negative effect. (4) Conclusions: The majority of nursing managers in various hospitals throughout the kingdom are creative and almost all use rational and dependent decision-making styles, which are significantly related to managerial creativity. Thus, it is important to continue to conduct training programs on decision-making styles, especially the rational, dependent, and avoidant styles, for the top-, middle-, and low-level managers.

## 1. Introduction

As a result of the widespread changes that are occurring today, it is recognized that health care organizations need creativity in order to succeed and survive. It is true that every organization’s success is greatly influenced by the effectiveness and efficiency of the decisions made, as well as their applicability to the goals established at various levels of management. This work explains the ways and reasons why people from various cultures occasionally have different decision-making patterns. Creativity is inspired by the saturation of solutions to a problem [1], and nursing managers spend the majority of their time studying problems and making decisions. The ability to integrate or combine ideas in a novel way to address long-standing organizational problems is necessary for creative decision making and the efficiency of an organization [2]. One of the most crucial tasks for managers in all kinds of organizations is making decisions. In all types of businesses, whether they are large or small, for profit or not for profit, private or public, it is a crucial managerial function [3].

Creativity and innovation are crucial to every institution’s growth and expansion. Promoting creativity among employees at all levels enhances the ability of the business to plan and implement changes from an organizational perspective. Managers make judgements to address issues, resolve crises, and solve difficulties. These choices are put into action to produce outcomes or results [4].

Analytical and intuitive decision-making methods are important. Both can be utilized to make efficient decisions [5]. Despite having confidence in their own ability, experienced nurses may not always make the best decisions [6]. Cultural awareness is closely related to the quality of decision making in Saudi Arabia, which in turn is closely related to experience. The obligation to make judgements outside of the scope of nursing in Saudi Arabia is one of the factors contributing to nurse frustration and job instability [7].

In nursing management, there are three functional roles: operational nurse manager (performs duties at the unit level), intermediate nurse manager (performs duties at the department level), and top-level nurse manager (performs his or her function at the organizational level) [8]. Top-level managers continue to make the vast majority of healthcare decisions in Saudi Arabia [9].

According to Flores-Garcia et al. [10], the literature on operations management presents various decision-making strategies regarding putting new process innovations into practice. Making normative decisions is one strategy. Normative decision making entails a quantitative assessment and a logical step-by-step examination, calling for information that is precise, objective, and well defined. Normative judgement, despite its purported limitations and difficulties, is nonetheless often utilized by companies and has frequently produced positive results [11].

When expert professionals are under time pressure, intuitive decision making might be helpful. However, it has several disadvantages. According to the literature, managers who rely on intuition may overlook key decision-making strategies regarding process innovation, struggle to articulate their decisions, or make egregious errors [10]. The involvement of managers in establishing and fostering environments that support the cognitive processes beneficial for developing creativity is even more significant [12].

Creativity can range from a low level to a high level. Lower-level creativity usually entails small-scale alterations and modifications of an existing idea or the new and beneficial integration of two or more previously unrelated ideas. Greater levels of creativity result in more ground-breaking contributions. The various forms of creativity include those that create new ideas, procedures, or concepts as well as those that alter pre-existing ones, combining previously unrelated ideas through creativity [13].

There are numerous and diverse styles that are used to make decisions according to the specific situation and time constraints. Creativity is an important skill for problem solving and developing new ideas for decision making. The question here is whether these styles affect the managerial creativity of nursing managers. This research is important to the management of government university hospitals in Saudi Arabia as it aids in understanding the impact of nursing managers with regard to their creativity and decision-making styles on the effectiveness of development operations. No single national or international study [14,15] focused on studying the relationship between managerial creativity and the decision-making style of nursing managers was found after reviewing the national and international literature related to this study topic. Thus, this study was conducted with the intention of focusing on this relationship.

### Aims of the Study

The present study was conducted to examine the relationship between decision-making styles and the managerial creativity of nursing managers.

## 2. Materials and Methods

### 2.1. Study Design and Participants

A multi-center cross-sectional design was used to carry out the present study. The study involved all nursing managers, comprising nurse directors, supervisors, and unit managers, working in a full-time position in five government university hospitals: King Fahad University Hospital, Al-Khobar; King Abdullah University Hospital, Riyadh; King Abdelaziz University Hospital and King Khalid Hospital, Riyadh; Najran University Hospital, Najran; and King Abdelaziz University Hospital, Jeddah. A cover letter describing the purpose of the study, informing participants of the voluntary nature of their participation, and assuring their data privacy was provided alongside the questionnaire.

### 2.2. Sampling Technique

This study used a non-probability sampling method (purposive sampling method), where the researcher selected the participants (nursing managers) to serve the overall objective of this study. The researcher used online surveys, which are an effective way to perform non-probability sampling. They are a quick and inexpensive method of data collection, in which nursing supervisors were asked to participate by answering an online questionnaire. The online link to the survey was sent to the nursing managers’ mobile number through the nursing director for each hospital. In order to prevent response duplication, the survey was tailored to only accept one response from each number.

### 2.3. Data Collection Tools and Processes

Data were collected using a validated online self-administered questionnaire consisting of two tools [16,17].

#### 2.3.1. Managerial Creativity Tool

The managerial creativity tool consists of two parts. The first part is a sociodemographic data questionnaire. This includes information such as age, gender, marital status, educational qualifications, job position, and years of experience. The second part is the managerial creativity questionnaire. Tawfiq [16] created this scale to assess nursing manager creativity. It consists of 35 items divided into seven subscales: Originality (five items), for instance, I perform the work assigned to me in a renewed manner and avoid repeating what others do to solve labor problems. Intellectual fluency (five items), for instance, I have the ability to suggest quick solutions to work problems. Flexibility (five items), for instance, I have the ability to present new ideas to develop the work automatically and conveniently. Sensitivity to problems (five items), for instance, I predict work problems before they happen. Maintaining direction and focus of attention (five items), for instance, I focus on my work tasks more than anyone else. Risk taking (five items), for instance, I accept criticism from others with open arms. Lastly, synthesis and analysis (five items), for instance, I have the ability to organize my ideas. The items were measured using a 5-point Likert scale ranging from 1 (strongly disagree) to 5 (strongly agree). The closer the answer is to 5, the more the respondent agrees with the stated question. The minimum and maximum scores are 35 and 175, respectively. The results are then categorized as the following: from 35 to 74 is considered below average creativity; from 75 to 125 is considered average creativity; and from 126 to 175 is considered very creative.

#### 2.3.2. General Decision-Making Style Questionnaire (GDMSQ)

The objective of the questionnaire is to evaluate nurse managers’ decision-making styles. There are 25 items in total, and they are divided into five decision styles: rational (five items), for instance, I confirm my source of information twice before believing it; dependent (five items), for instance, I often need help from other people while making decisions; avoidant (five items), for instance, I postpone important decisions until there is pressure; intuitive (five items), for instance, I rely upon my instincts while making a decision; and lastly, spontaneous (five items), I generally make decisions instantly. Using a 5-point Likert scale, the responses were rated from 1 (strongly disagree) to 5 (strongly agree).

Each of the five GDMSs had individual item scores that were added together to produce values ranging from 5 to 25 for each style. Each nurse manager’s score was transformed into a percentage score. A high level was defined as a percentage score equal to or greater than 75%; a moderate level was defined as a percentage score of 60% to 75%; and a low level was defined as a percentage score of less than 60%.

### 2.4. Validity and Reliability

To evaluate the tool’s clarity, understandability, and applicability; estimate the time taken for the study; and highlight potential issues that could arise during data collection, a pilot study was conducted. The pilot study was applied to 10% of the overall sample of nurse managers (n = 25). No adjustments were made to the study tools after the data from the pilot study were analyzed. The study tools’ reliability was evaluated using a Cronbach’s alpha-coefficient test, which resulted in scores of 0.903 on the management creativity scale and 0.724 on the general decision-making style scale. Thus, it was evident that the study tools are highly reliable.

### 2.5. Study Procedure

Preparatory phase: This phase took about four months, from August to November 2022, to conduct a review of the available literature concerning the topic of the study. In addition, a research proposal was made in order to obtain ethical approval from the authorization committee. Then, a list of eligible nurses was obtained from the nursing human resource liaison manager. At this time, the online link for the questionnaire was sent to the nurses via phone through the nursing manager. Before collecting data, informed consent was obtained. Finally, the respondents were asked to return the signed consent form and completed questionnaire within 1 week upon receiving the link. The questionnaires took about fifteen minutes to complete for each participant. Actual data collection was conducted in February 2023.

### 2.6. Statistical Analysis and Data Management

Data entry and statistical analyses were performed using Statistical Package for the Social Sciences, version 22.0 (IBM Corp., Armonk, NY, USA). The statistics were tested for normality via the Anderson–Darling test and for homogeneity before additional statistical analyses. Data were presented using descriptive statistics in the form of frequencies, percentages, means, standard deviations, ranges, Chi-square, and multivariate linear regression. A Pearson correlation analysis was used for assessment of the interrelationships among quantitative variables. Statistical significance was considered at a *p*-value of ≤0.05.

### 2.7. Ethical Approval

The study proposal was reviewed and approved under Imam Abdulrahman bin Faisal University IRB number 2022-01-537. Prior to commencing data collection, all participants were informed of the study’s objective, nature, and aim, and the confidentiality of their data was guaranteed. The contributors were notified about their rights to privacy and that they could contribute or withdraw at any time during the study. Next, their consent was obtained. Their informed consent was confirmed.

## 3. Results

Table 1 depicts the distribution of nurses based on their sociodemographic data. In terms of age, almost half (46.9%) of nursing managers were 30–40 years old, 42.0% were less than 30 years old, and 11.0% were more than 40 years old. More than half (57.1%) worked at King Abdelaziz University Hospital, 13.9% worked at King Fahd University Hospital, 13.1% worked at King Abdullah University Hospital, 6.5% worked at King Khalid Hospital, and 9% worked at Najran University Hospital. As regards gender, the majority (97.6%) of them were female, most (84.1%) of them were married, and a minority (14.7%) were single. In terms of education, the majority (89.4%) of them held bachelor’s degrees in nursing. About half (50.6%) of them occupied middle-management positions, 42.4% were in a low-level management position, and 6.9% held a top management position. More than two-fifths (43.7%) of them had experience spanning more than 10 years and more than one-third (36.7%) of them had experience ranging from 5 to 10 years.

Table 2 illustrates that the rational style had the highest mean score (20.95 ± 2.03, 83.8%) and the widest range (15–25), followed by the dependent style (17.01 ± 2.8, 68.04%) with a range of 11–23 and the avoidant style (12.8 ± 2.88, 51.2%) with the lowest mean score and a range of 7–21.

Regarding the managerial creativity subdomains, synthesis and analysis had the highest mean score (22.55 ± 2.19, 90.2%) and the widest range (17–25), followed by maintaining focus and direction (21.76 2.04, 87.04%) with a range of 16–25 and originality (20.95 ± 1.8, 83.8%) with the lowest mean score and a range of 16–25.

Table 3 reveals the relationship between decision-making styles and managerial creativity (*p* = 0.001 **). The data demonstrate that there are highly statistically significant differences between rational, avoidant, and dependent decision-making styles and the overall managerial creativity of nursing managers. No statistically significant differences were found between intuitive and spontaneous styles and overall managerial creativity. In detail, there were highly statistically significant differences between the rational decision-making style and all managerial creativity subdomains (intellectual fluency, flexibility, sensitivity to problems, maintaining direction and focus of attention, and risk taking) with *p* values of 0.001 **, 0.001 **, 0.001 **, 0.001 **, and 0.001 **, respectively, except for synthesis and analysis. Additionally, there were highly statistically significant differences between the avoidant decision-making style and all managerial creativity subdomains, except for the originality subdomain, with a *p* value of 0.215. Moreover, there were highly statistically significant differences between the dependent decision-making style and all managerial creativity subdomains, except for maintaining direction and focus of attention, with only a statistically significant difference of 0.014 *, and flexibility (0.081), where there was no statistically significant difference. There was a highly statistically significant difference between the intuitive decision-making style and risk-taking subdomain (0.001 **). Finally, there were statistically significant differences between the spontaneous decision-making style with both sensitivity to problems and synthesis and analysis (0.001 ** and 0.033 *, respectively).

Table 4 demonstrates a strong significant correlation between rational style and total managerial creativity (r = 0.478; *p* = 0.001 **) and across all managerial creativity subdomains. However, there is a strong significant inverse correlation between all other styles of decision making (avoidant, dependent, and spontaneous) and managerial creativity (r = −0.385; *p* = 0.001 **, r = −0.310; *p* = 0.001 **, and r = −0.214; *p* = 0.001 **, respectively), except for the intuitive style (r = −0.032; *p* = 0.617).

As shown in Table 5, three styles of decision making had a significant effect on managerial creativity; the rational style had the highest effect (β = 0.388; *p* = 0.001 **), followed by the dependent style (β = −0.251; *p* = 0.001 **) and finally the avoidant style (β = −0.188; *p* = 0.001 **). It is noted that neither the intuitive nor the spontaneous decision-making styles had a significant effect on managerial creativity. 

In Table 6, no significant differences were observed between decision-making style and all socio-demographic data, except for intuitive and spontaneous styles and the place of work (*p* = 0.001 ** and *p* = 0.007 **, respectively). King Fahd University Hospital had the highest mean scores for both styles (18.12 ± 2.57 and 15.32 ± 3.3, respectively).

As shown in Table 7, significant differences in managerial creativity were observed among all management levels, places of work, and educational qualifications (*p* = 0.003 **, *p* = 0.016 *, and *p* = 0.047 *, respectively). There were no significant differences between managerial creativity and age, gender, marital status, or experience (0.061, 0.570, 0.339, and 0.135, respectively).

**Figure 1:**
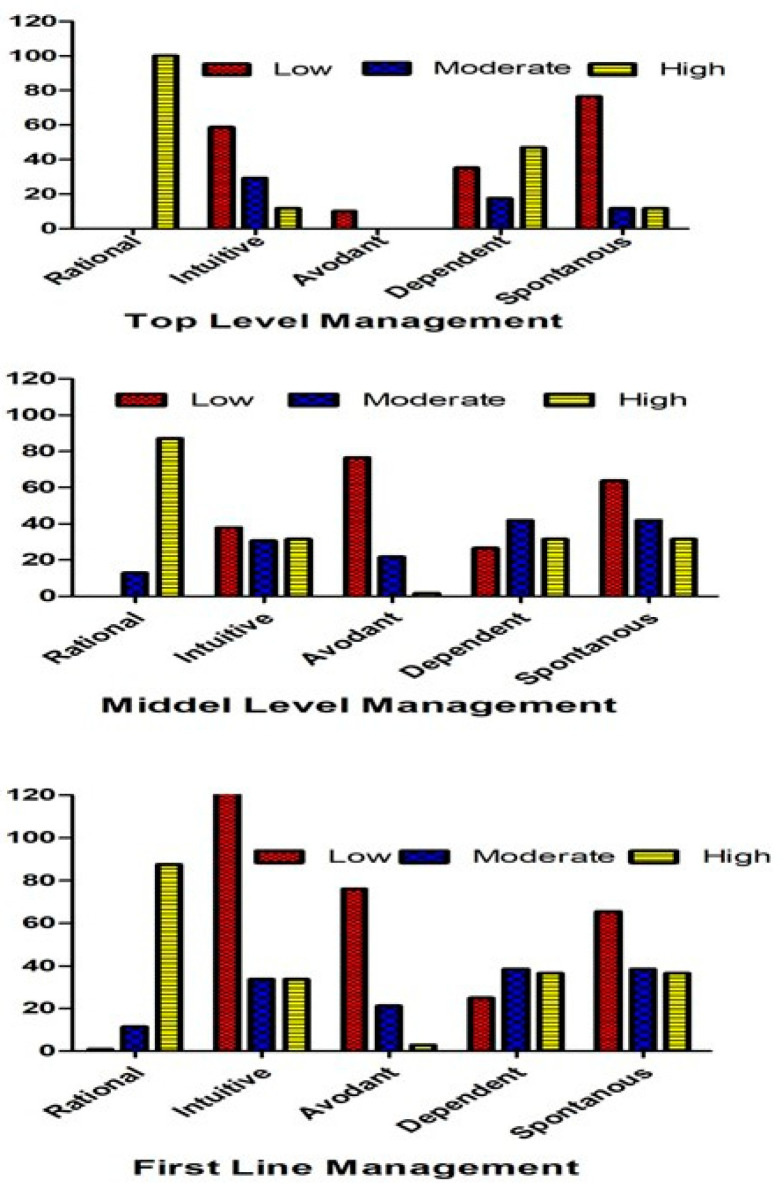
Decision-making styles according to three management levels.

Figure 1 shows that top-level nursing managers had strong links with both rational and dependent decision-making styles, with percentages of 100% and 47.1%, respectively. Intuitive decision making was used at a moderate (58.8%) level and spontaneous decision making was used at a low level.

Regarding middle-level nursing managers, they were highly likely (87.1%) to use rational decision-making styles, moderately likely (41.9%) to use dependent decision-making styles, and least likely (76.6% and 63.7%) to use avoidant and spontaneous decision-making styles, respectively.

Low-level managers were highly likely (87.5%) to use rational decision-making styles, moderately likely (38.5%) to use dependent decision-making styles, and least likely (76.3%) to use avoidant decision-making styles. 

**Figure 2:**
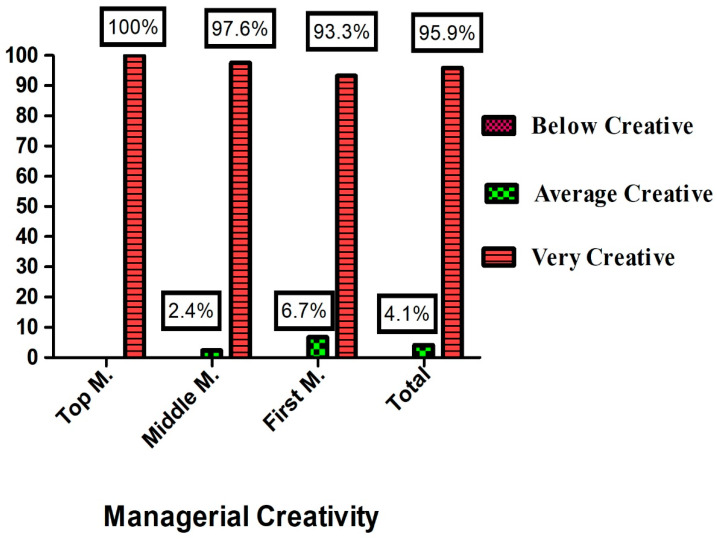
Levels of managerial creativity for studied nurses (N = 245).

Figure 2 shows that the nursing managers in general were very creative (95.9%). However, only 4.1% of them were averagely creative, and no one was below average in creativity. Regarding the three management positions, it was observed that top-level management had the highest score (100%), followed by middle-level management (97.6%) and finally low-level management (93.3%). The results demonstrate that all levels of management were assessed as very creative.

## 4. Discussion

This study is one of the few that aim to examine the relationship between decision-making styles and managerial creativity among nursing managers in Saudi Arabia. The main findings of this study revealed that the majority of nursing managers used rational and dependent styles of decision making in all three management levels. Additionally, the majority of them were very creative in all levels of management. Additionally, a regression analysis revealed that the rational style had the highest and only positive effect on managerial creativity. Although dependent and avoidant styles were the second and third most effective on managerial creativity, their impacts were negative.

The findings revealed that a strong correlation is present between the rational style and overall managerial creativity. Additionally, a high percentage of the studied nursing managers were very creative in all three management levels. The managerial creativity of nursing managers across different hospitals was affected mostly by the rational decision-making style and also by the dependent style (they had the highest mean scores). This agrees with the work of Ali and Elhakem [18], who discovered that office executives from different departments possessed a high degree of creativity, and directors in administration-related fields are the most likely to adopt the rational style when making decisions. The determined increased utilization of the rational style corresponds emphatically with the work of Lamba and Ozdasli [19]. Additionally, Panatik [20] found in their study that Malaysian research officers had a high level of creativity. The results supported the findings of Riaz [21], showing that those who employ the rational approach do not hesitate to do so when coming to a conclusion.

This study showed that the most important managerial creativity elements perceived by nursing managers are ranked in descending order as synthesis and analysis, maintaining direction and focus of attention, intellectual fluency, risk taking, sensitivity to problems, flexibility, and originality. This finding is partially consistent with the findings of Ali and Elhakem [18], which reported that the most important managerial creativity elements available to managers in Sudanese institutions are ranked in descending order as synthesis and analysis, maintaining direction, risk taking, flexibility, originality, intellectual fluency, and problem sensitivity. 

The data collected in this study demonstrated that managerial creativity was negatively affected when nursing managers adopt avoidant, dependent, and spontaneous styles of decision making. According to Elrais and Mohamed [22], the majority of nurse managers tend to make decisions in independent and moderately spontaneous ways. They adopt an avoidant manner in the meantime. According to Al Shra’ah [23], the decision-making style of any manager or in any institution depends on competence and learning processes, in which academic achievement was thought to be a significant component in comprehending the decision-making process [24]. On the other hand, this conclusion conflicts with a report from Slosky et al. [25], who noted that engagement in decision making was unrelated to academic qualifications.

In this study, the majority of nursing managers had a bachelor’s degree and more than two-fifths of them had more than ten years of experience. According to Manlangit et al. [26], in Saudi Arabia, the effectiveness of decision making is strongly correlated with experience.

Furthermore, avoidant and spontaneous styles have been shown to predict decision-making abilities, where the intuitive style has been shown to be a positive factor and the avoidant style a negative factor [4]. Despite the fact that people frequently utilize many different decision-making styles, the relationships between decision-making styles highlight those who have a dominating style.

Saleh et al. [27] claim that for a little more than half of nurse managers, creativity is above average, as assessed in top-, middle-, and low-level nursing managers. However, Faizanul [28] claimed in their study titled “Creativity is the Key to Success” that most individuals are not ready to change their thinking because they lack the bravery to take chances and are reluctant to change.

The present study revealed a positive statistically significant relationship between managerial creativity and workplace (Table 6), especially for Najran University Hospital and King Abdelaziz University Hospital, which have the highest mean scores. There was also a positive statistically significant relationship between managerial creativity and occupation, especially in top-level management positions, and lastly between managerial creativity and educational level, especially for postgraduates. According to these findings, a higher educational level and management position increase creativity and vice versa. This result is inconsistent with work by Saleh et al. [27], who reported that there was a negative correlation between leaders’ qualification levels and creativity.

The avoidant style is characterized by delay and denial, as stated by Scott and Bruce [17]. According to Hossny et al. [29,30], managers can decide and create appropriate approaches to cut down on time wasted at work and promote sustainability. Additionally, in order to solve problems that cause crises, fresh ideas and inventive solutions are needed.

This study is the first to examine the relationship between decision-making styles and managerial creativity of nursing managers in Saudi Arabia. The study also included the largest hospitals in the province in the Kingdom of Saudi Arabia (multi-center). An online survey was used to recruit a large number of participants. Moreover, all the Nursing managers of the same University Hospital are included. However, despite these strengths, the current study has some limitations. The current study was a cross-sectional survey. Therefore, may indicate bias. In addition, nationality was not included in the study, so in future studies, the author proposes to analyze the nationality of the study participants.

## 5. Conclusions

The results obtained showed that there was a strong positive link between managerial creativity and rational decision-making styles. The rational style had the highest effect on managerial creativity, followed by avoidant and dependent styles. The majority of nursing managers in various hospitals throughout the kingdom are creative and almost all used rational and dependent decision-making styles, which were significantly related to managerial creativity. The managers in the organization’s top, middle, and low levels are better suited to the rational decision-making style. The percentages of dependent and rational decision making in top management are the highest. Significant negative correlations were found between avoidant, independent, and spontaneous decision-making styles and managerial creativity.

Thus, top-, middle-, and low-level nursing managers should participate in ongoing training programs on decision making, particularly on rational, dependent, and avoidant styles, to highlight their significance and consequences for creativity.

## Figures and Tables

**Table 1:** Distribution of socio-demographic data for study nursing managers (N = 245).

Socio-Demographic Data	(No)	(%)
**Age group**		
Less than 30	103	42.0
30–40	115	46.9
More than 40	27	11.0
M ± SD (range)	33.95 ± 6.30 (25–59)	
**Place of work**		
King Fahd University Hospital	34	13.9
King Abdullah University Hospital	32	13.1
King Khalid Hospital	16	6.5
Najran University Hospital	23	9.4
King Abdelaziz University Hospital	140	57.1
**Gender**		
Male	6	2.4
Female	239	97.6
**Marital Status**		
Single	36	14.7
Married	206	84.1
Divorce	3	1.2
**Education Level**		
Nursing bachelor	219	89.4
Postgraduate	23	9.4
Nursing diploma	3	1.2
**Job positions**		
Top Management	17	6.9
Middle Level	124	50.6
First Level	104	42.4
**Years of Experience**		
Less than 5 years	48	19.6
5–10 years	90	36.7
More than 10 years	107	43.7
M ± SD (range)	11.27 ± 6.35(2–35)	

Note: mean (M), standard deviation (SD), and sub sample (n).

**Table 2:** Max score, mean and standard deviations, and range of decision-making and managerial creativity styles of the studied nurses (N = 245).

Study Variables	Max Score	M ± SD (%)	Range
**Decision-making style**			
Rational	25	20.95 ± 2.03 (83.8)	15–25
Dependent	25	17.01 ± 2.8 (68.0)	11–23
Intuitive	25	16.54 ± 2.84 (66.1)	9–23
Spontaneous	25	14.23 ± 3.09 (56.9)	8–22
Avoidant	25	12.8 ± 2.88 (51.2)	7–21
**Managerial creativity subdomains**			
Synthesis and analysis	25	22.55 ± 2.19 (90.2)	17–25
Maintaining direction and focus of attention	25	21.76 ± 2.04 (87.0)	16–25
Intellectual fluency	25	21.6 ± 2.13 (86.4)	17–25
Risk taking	25	21.61 ± 1.87 (86.4)	17–25
Sensitivity to problems	25	21.27 ± 2.3 (85.0)	15–25
Flexibility	25	21.26 ± 2.16 (85.0)	14–25
Originality	25	20.95 ± 1.8 (83.8)	16–25
**Total Managerial Creativity**	175	150.99 ± 10.19 (86.2)	125–173

Note: mean (M), standard deviation (SD), percent (%).

**Table 3:** Relation between decision-making styles and managerial creativity of nursing managers (N = 245).

	DMS	Rational		Intuitive		Avoidant		Dependent		Spontaneous	
MC		M ± SD	*p* Value	M ± SD	*p* Value	M ± SD	*p* Value	M ± SD	*p* Value	M ± SD	*p* Value
Originality		20.9 ± 1.7	0.211	21.2± 2.02	0.290	19.6 ± 2.51	0.215	21.11 ± 1.53	0.001 **	21.27 ± 1.56	0.537
Intellectual fluency		21.7 ± 2.12	0.001 **	21.7 ± 1.95	0.025	20.2 ± 1.1	0.005 **	21.29 ± 2.27	0.001 **	21.77 ± 2.07	0.742
Flexibility		21.4 ± 2.07	0.001 **	20.8 ± 2.47	0.104	18.6 ± 4.67	0.009 **	21.02 ± 2.37	0.081	20.62 ± 3.7	0.120
Sensitivity to problems		21.4 ± 2.11	0.001 **	21.4 ± 2.76	0.101	20 ± 1.22	0.001 **	20.55 ± 2.85	0.001 **	19.77 ± 3.43	0.001 **
Maintaining direction and focus of attention		22.0 ± 1.8	0.001 **	21.7 ± 2.3	0.502	21.8 ± 1.79	0.003 **	21.45 ± 2.43	0.014 *	21.15 ± 3.38	0.222
Risk taking		21.8 ± 1.71	0.001 **	21.4 ± 1.94	0.001 **	21.6 ± 2.41	0.001 **	21.19 ± 2.17	0.003 **	21.27 ± 2.54	0.606
Synthesis and analysis		22.7 ± 2.1	0.003 **	22.2 ± 2.55	0.433	21.8 ± 2.05	0.001 **	21.91 ± 2.59	0.001 **	21.54 ± 3.11	0.033 *
Overall Managerial Creativity		152.2 ± 9.19	0.001 **	150.7 ± 10.9	0.084	143.6 ± 10.71	0.001 **	148.52 ± 11.3	0.001 **	147.38 ± 15.12	0.160

Note: mean (M), standard deviation (SD). * Significant level at *p* value 0.05. ** Significant level at *p* value 0.01.

**Table 4:** Correlation co-efficient between decision-making styles and managerial creativity subdomains.

	MC	Rational		Intuitive		Avoidant		Dependent		Spontaneous	
DMS		r	*p*	R	*p*	R	*p*	r	*p*	r	*p*
Originality		0.135	0.034 *	0.049	0.442	−0.122	0.056	−0.132	0.039 *	−0.100	0.119
Intellectual fluency		0.314	0.001 **	0.008	0.905	−0.323	0.001 **	−0.250	0.001 **	−0.133	0.037 *
Flexibility		0.264	0.001 **	−0.071	0.266	−0.182	0.004 **	−0.133	0.038 *	−0.082	0.203
Sensitivity to problems		0.397	0.001 **	−0.026	0.681	−0.347	0.001 **	−0.311	0.001 **	−0.240	0.001 **
Keep direction and focus of attention		0.457	0.001 **	−0.020	0.751	−0.254	0.001 **	−0.193	0.002 **	−0.121	0.058
Risk taking		0.402	0.001 **	−0.109	0.090	−0.313	0.001 **	−0.255	0.001 **	−0.150	0.019 *
Synthesis and analysis		0.363	0.001 **	0.012	0.855	−0.328	0.001 **	−0.236	0.001 **	−0.211	0.001 **
Total (MC)		0.478	0.001 **	−0.032	0.617	−0.385	0.001 **	−0.310	0.001 **	−0.214	0.001 **

* Statistically significant correlation at a *p* value of 0.05, ** Statistically significant correlation at a *p* value of 0.01.

**Table 5:** Multivariate linear regression between decision-making styles and managerial creativity.

Managerial Creativity				
Decision-Making Style	B	β	T	*p* Value
Rational	1.950	0.388	6.007	0.001 **
Intuitive	0.341	0.095	1.570	0.118
Avoidant	−0.664	−0.188	−2.391	0.018 *
Dependent	−0.913	−0.251	−4.249	0.001 **
Spontaneous	0.367	0.111	1.440	0.151

Note: The dependent variables are managerial creativity, unstandardized beta (B), standardized beta (β), the *t*-test statistic (t), and the probability value (*p*). * Significant factor at a *p* value of 0.05 ** significant factor at a *p* value of 0.01.

**Table 6:** Relationship between decision-making styles of the studies’ nurses and socio-demographic data.

Socio-Demographic Data	Decision-Making Style				
	Rational	Intuitive	Avoidant	Dependent	Spontaneous
	**M ± SD**	**M ± SD**	**M ± SD**	**M ± SD**	**M ± SD**
**Age group**					
Less than 30	20.93 ± 2.13	16.83 ± 2.75	12.92 ± 3.08	17.28 ± 2.74	14.31 ± 3.03
30–40	20.8 ± 1.96	16.32 ± 3.02	12.85 ± 2.81	16.67 ± 2.85	14.28 ± 3.14
More than 40	21.63 ± 1.84	16.37 ± 2.31	12.07 ± 2.27	17.44 ± 2.74	13.7 ± 3.18
*p* value	0.160	0.405	0.381	0.190	0.645
**Place of work**					
King Fahd University Hospital	20.97 ± 2.26	18.12 ± 2.57	13.18 ± 3.23	17.47 ± 2.58	15.32 ± 3.3
King Abdullah University Hospital	20.94 ± 1.81	16.34 ± 2.16	12.88 ± 2.78	16.63 ± 3.11	13.63 ± 2.83
King Khalid Hospital	20.31 ± 1.82	16.63 ± 2.19	13.31 ± 2.65	17.13 ± 3.1	15.25 ± 2.72
Najran University Hospital	21.78 ± 1.68	14.13 ± 2.67	11.48 ± 2.43	16.17 ± 2.84	12.57 ± 2.84
King Abdelaziz University Hospital	20.88 ± 2.08	16.59 ± 2.88	12.84 ± 2.88	17.11 ± 2.73	14.26 ± 3.06
*p* value	0.229	0.001 **	0.200	0.436	0.007 **
**Gender**					
Male	21.83 ± 2.4	16.67 ± 4.37	11.67 ± 2.34	15.67 ± 3.98	13.5 ± 2.74
Female	20.92 ± 2.02	16.54 ± 2.8	12.82 ± 2.89	17.05 ± 2.76	14.25 ± 3.1
*p* value	0.279	0.911	0.332	0.234	0.560
**Education Level**					
Nursing Bachelor	20.92 ± 2.08	16.55 ± 2.88	12.79 ± 2.97	16.96 ± 2.81	14.26 ± 3.13
Postgraduate	21.35 ± 1.58	16.26 ± 2.22	12.91 ± 1.9	17.39 ± 2.74	13.83 ± 2.66
Nursing diploma	20 ± 1	18 ± 4.36	12 ± 2.65	18 ± 2.65	14.67 ± 4.04
*p* value	0.452	0.603	0.876	0.647	0.788
**Occupation**					
Top Management level	22 ± 1.66	15.71 ± 2.11	11.76 ± 2.11	17.29 ± 2.95	12.76 ± 3.51
Middle Level	20.9 ± 1.95	16.47 ± 2.94	12.89 ± 2.79	16.85 ± 2.83	14.44 ± 3.07
Low Level	20.84 ± 2.14	16.76 ± 2.81	12.86 ± 3.08	17.15 ± 2.75	14.22 ± 3.01
*p* value	0.083	0.339	0.310	0.662	0.112
**Years of Experience**					
Less than 5 years	20.73 ± 2.09	17.4 ± 2.64	13.13 ± 3.32	17.65 ± 2.29	14.67 ± 3.2
5–10 years	20.96 ± 2.1	16.3 ± 2.93	12.78 ± 2.8	16.87 ± 3.04	14.01 ± 2.82
More than 10 years	21.04 ± 1.95	16.36 ± 2.8	12.66 ± 2.75	16.85 ± 2.77	14.21 ± 3.26
*p* value	0.683	0.065	0.653	0.217	0.495

Independent *t*-test between the two groups, one-way ANOVA *t*-test between the three groups or more, ** Significant level at a *p* value of 0.01.

**Table 7:** Relationship between socio-demographic data and managerial creativity and decision-making style.

Socio-Demographic Data	Managerial Creativity			*p* Value
	N	M ± SD	Range	
**Age group**				
Less than 30	103	150.1 ± 10.7	125–173	0.061
30–40	115	150.79 ± 9.81	125–173	
More than 40	27	155.26 ± 9.01	129–169	
**Place of work**				
King Fahd University Hospital	34	148.06 ± 10.83	125–166	0.016 *
King Abdullah University Hospital	32	149.28 ± 10.21	125–169	
King Khalid Hospital	16	150.63 ± 9.44	133–169	
Najran University Hospital	23	157.09 ± 8.26	138–173	
King Abdelaziz University Hospital	140	151.14 ± 10.1	125–173	
**Gender**				
Male	6	153.33 ± 17.33	129–173	0.570
Female	239	150.93 ± 10	125–173	
**Education Level**				
Nursing Bachelor	219	150.58 ± 10.35	125–173	0.047 *
Postgraduate	23	155.65 ± 6.66	138–169	
Nursing diploma	3	145.33 ± 14.15	129–154	
**Occupation**				
Top Management level	17	158.94 ± 5.4	148–169	0.003 **
Middle Level	124	150.2 ± 10.05	125–173	
Low Level	104	150.63 ± 10.47	125–173	
**Years of Experience**				
Less than 5 years	48	148.52 ± 10.62	125–164	0.135
5–10 years	90	151.03 ± 10.42	125–173	
More than 10 years	107	152.07 ± 9.7	125–172	

Independent *t*-test between the two groups one-way ANOVA *t*-test between the three groups or more. * Significant level at a *p* value of 0.05, ** Significant level at a *p* value of 0.01.

## Data Availability

The data in the current study are available upon request from the corresponding author.
